# Impact of insurance on outcomes of patients undergoing endoscopic transsphenoidal surgery for non-functional pituitary adenomas: a single institution study

**DOI:** 10.1007/s11102-024-01478-w

**Published:** 2024-12-30

**Authors:** Khushi H. Shah, Nikola Susic, Adham M. Khalafallah, Victor M. Lu, Michael E. Ivan, Ricardo J. Komotar, Zoukaa B. Sargi, Ashish H. Shah

**Affiliations:** 1 Department of Neurological Surgery, Miller School of Medicine, University of Miami, Miami, FL USA; 2 Department of Otolaryngology, Miller School of Medicine, University of Miami, Miami, FL USA; 3 Sylvester Comprehensive Cancer Center, University of Miami Health System, Miami, FL USA; 4 Department of Neurological Surgery, Miller School of Medicine, University of Miami, 1475 NW 12th Ave, Miami, FL 33136 USA

**Keywords:** Pituitary adenoma, Insurance, Outcome, Endoscopic transsphenoidal pituitary surgery

## Abstract

**Purpose:**

Uninsured and underinsured patients face notable healthcare disparities in neurosurgery, but limited literature exists on the impact of insurance on non-functioning pituitary adenomas (NFPAs). We investigated how insurance affects outcomes of endoscopic transsphenoidal pituitary surgery (ETPS) for NFPAs.

**Methods:**

We retrospectively reviewed NFPA patients who underwent ETPS at our institution from 2012 to 2023. Patients were grouped by insurance status, and insured patients were further subcategorized by insurance providers. Bivariate analyses used Fisher’s exact, chi-square, and t-tests. ANOVA or Kruskal-Wallis tests were applied for ≥ 2 groups. Logistic regression identified relationships between binomial variables and insurance.

**Results:**

Our cohort (*n* = 651, 56.93 ± 15.53 years, 52.53% male) included 611 insured and 40 uninsured patients. Uninsured patients had lower preoperative KPS, higher rates of visual disturbances (VD), preoperative tumor volumes (TV), chiasm compression, and Knosp 4 grade, along with lower resection and longer hospital stays (LOS) (*p* < 0.05). Multivariate analysis showed lack of insurance was associated with increased VD (aOR 3.38), TV (aOR 2.63), Knosp 4 (aOR 3.44), subtotal resection (aOR 2.72), and prolonged LOS (aOR 7.03) (*p* < 0.05). When insured patients were grouped into Private (*n* = 361), Medicare (*n* = 223), and Medicaid (*n* = 23), Medicaid patients had larger preoperative TV, chiasm compression, Knosp 3 grade, and longer LOS (*p* < 0.05), with higher odds for Knosp 3 (aOR 3.00), subtotal resection (aOR 3.86), and prolonged LOS (aOR 8.38) (*p* < 0.05).

**Conclusion:**

Our study highlights significant disparities in uninsured patients and those with Medicaid, underscoring the need for targeted interventions for these populations.

## Introduction

It is well known that socioeconomic disparities influence patients’ access to health care and significantly impact outcomes following neurosurgical procedures [1–4]. Insurance status is an important socioeconomic factor and is considered a surrogate marker of social determinants of health [5]. Previous studies have demonstrated that uninsured patients and those with Medicaid often present with worse disease severity, experience increased length of hospital stay (LOS), and have a higher rate of postoperative complications following craniotomy and spine surgery [6, 7]. These factors translate to worse outcomes and higher mortality after neurosurgical procedures [6, 7].

Pituitary adenomas (PAs), although typically benign, pose a significant burden to patients and the healthcare system [8–10]. Several studies have highlighted the impact of socioeconomic factors on outcomes of endoscopic transsphenoidal surgery (ETPS) for PA [11, 12]. However, to our knowledge, there are only two studies that have specifically focused on the impact of insurance on patients with PAs, covering both functional PAs (FPAs) and non-functional PAs (NFPAs) in their cohorts [13, 14]. Given the differences in presentation, diagnosis, and treatments between FPAs and NFPAs, it is important to refine existing literature by elucidating the impact of insurance in each group specifically. Although the Affordable Care Act broadened insurance coverage [15], our experience at a large academic institution in an urban setting reveals ongoing challenges treating uninsured and underinsured patients.

There is little research focused on how insurance impacts outcomes of patients with NFPA. Therefore, in this study, we investigated the effect of insurance status and provider on outcomes of patients undergoing ETPS for NFPA. Elucidation of these disparities will aid in empowering neurosurgeons to initiate actionable changes to equilibrate future outcomes [1].

## Methods

### Patient

After Institutional Review Board Approval (IRB no. 20160437), a retrospective chart review was conducted of patients who underwent ETPS at our tertiary care institution from 2012 to 2023. Patients were evaluated preoperatively with magnetic resonance imaging (MRI) and hormonal workup. Surgical technique [16] and postoperative management [17] are described previously. Patients with age ≥ 18 years, NFPA as identified by presentation and preoperative hormonal workup, and histopathological confirmation of PA were included in this study. Patients with FPA were excluded. Informed consent was waived due to retrospective nature of study.

### Data collection

Patient demographics and preoperative data, including age, gender, insurance status, comorbidities, preoperative Karnofsky performance score (KPS), visual disturbances (VD) as determined by neurological exam at admission as well as ophthalmological exams from prior follow-ups or ophthalmology consults, apoplexy, history of radiation to the pituitary, were collected. MRI scans were reviewed for preoperative tumor volume (TV), chiasm compression, macroadenoma (tumor diameter > 10 mm), and the probability of cavernous sinus invasion as determined by the Knosp-Steiner classification. TVs were calculated using the ellipsoid formula (length x width x height/2), based on the greatest dimensions in axial, coronal, and sagittal planes from MRI scans. Intraoperative data included CSF leak and lumbar drain use. Residual TV and extent of resection (EOR) were calculated through 24-hour postoperative MRI scans. Gross total resection (GTR) was defined as EOR equal to 100%. Data on outcomes included LOS, postoperative complications including CSF leak, diabetes insipidus (DI), hyponatremia, epistaxis, hyposmia, septal perforation, deep vein thrombosis, and vascular injury, 30-day readmission, and tumor recurrence.

### Statistical analysis

Patients were categorized based on their insurance status (insured vs. uninsured) at the time of surgery. Insured patients were further categorized by type of insurance: Private, Medicare, Medicaid, and TRICARE. Categorical variables were analyzed using chi square or Fisher exact tests. For comparison of continuous variables between 2 groups, Student’s t-test or Welch’s t-tests were used depending on the equality of variance tested via Levene’s test. For comparison of continuous variables between ≥ 2 groups, ANOVA or Kruskal-Wallis tests were used, followed by Tukey or pairwise Wilcox tests for significant comparisons. Patients with TRICARE were excluded from insurance subgroup analysis due to low sample size (*n* = 4). Mean and standard deviation were reported for all continuous variables, except for preoperative KPS and LOS, where median and interquartile range (25-75th percentile) were used due to non-normal distribution.

Univariate analysis via logistic regression with cross validation was conducted to examine relationships between binomial outcome variables and insurance status. Linear variables were converted to binomial format for this analysis. Prolonged LOS (PLOS), defined as 90th percentile of LOS [18], was compared with standard LOS. TVs were divided into 2 groups based on the median TV and EOR was analyzed as gross total resection (GTR) or subtotal resection (STR). Variables were then assessed in a multivariate model controlling for age, gender, and preoperative KPS to obtain an adjusted odds ratio (aOR). Statistical analyses were performed using Python version 3.11.5 for Windows.

## Results

### Insurance status: descriptive statistics

During the study period, out of 1070 patients who underwent ETPS at our institution, 651 patients met the inclusion criteria. Of these, 611 (mean age 57.37 ± 15.56 years, 52.70% male) were insured and 40 (mean age 49.12 ± 13.80 years, 50.00% male) were uninsured. There was no significant difference in gender, comorbidities, history of radiation, and apoplexy between groups (Table 1). Insured patients were significantly older (*p* ≤ 0.001). Uninsured patients had significantly lower preoperative KPS compared to insured patients (85 [80–90] vs. 90 [80–90], *p* = 0.004). Uninsured patients had higher rates of VD (82.50% vs. 55.81%, *p* = 0.002) and chiasm compression (87.18% vs. 75.41%, *p* = 0.009), higher preoperative TV (13.68 ± 16.89 vs. 6.62 ± 10.27 cm^3^, *p* = 0.005), and Knosp 4 grade (22.50% vs. 8.35%, *p* = 0.007).

**Table 1:** Patient demographics, clinical, and perioperative characteristics for insured vs. uninsured group

Variable	Insured (*n* = 611)	Uninsured (*n* = 40)	*p* value
**Patient demographics:**			
Age (years), mean, SD	57.37 ± 15.56	49.12 ± 13.80	**< 0.001**
Gender, male	322 (52.70%)	20 (50.00%)	0.867
Preop KPS, median, IQR	90 (80–90)	85 (80–90)	**0.004**
BMI, mean, SD	29.48 ± 6.14	30.91 ± 10.00	0.447
Diabetes mellitus	173 (28.31%)	11 (27.50%)	1.000
Hypertension	326 (53.35%)	24 (60.00%)	0.514
Active smoker at time of surgery	46 (7.53%)	2 (5.00%)	0.760
Hx of radiation	6 (0.98%)	0 (0.00%)	1.000
VD	341 (55.81%)	33 (82.50%)	**0.002**
Apoplexy	38 (6.22%)	4 (10.00%)	0.317
**Adenoma characteristics**:			
Chiasm compression	460 (75.41%)	34 (87.18%)	**0.009**
Macroadenoma	576 (94.27%)	37 (92.50%)	0.502
Knosp grades:			
Grade 0	98 (16.20%)	10 (25.00%)	0.221
Grade 1	137 (22.42%)	6 (15.00%)	0.367
Grade 2	110 (18.00%)	4 (10.00%)	0.281
Grade 3	214 (35.02%)	11 (27.50%)	0.425
Grade 4	51 (8.35%)	9 (22.50%)	**0.007**
Preop TV (cm^3^), mean, SD	6.62 ± 10.27	13.68 ± 16.89	**0.005**
**Operative characteristics**:			
Intraop CSF leak	333 (54.50%)	27 (70.00%)	0.081
Lumbar drain	13 (2.13%)	1 (2.50%)	0.592
Residual TV (cm^3^), mean, SD	0.35 ± 1.43	2.85 ± 7.18	**0.011**
EOR (%), mean, SD	96.46 ± 9.60	85.44 ± 26.03	**0.017**
**Treatment outcomes**:			
LOS (days), median, IQR	3 (3–4)	5 (3–7)	**< 0.001**
Overall complications	354 (57.93%)	27 (67.50%)	0.306
Postop CSF leak	20 (3.27%)	2 (5.00%)	0.639
Transient DI	280 (45.83%)	20 (50.00%)	0.727
Permanent DI	67 (10.96%)	6 (15.00%)	0.576
Hyponatremia	70 (11.46%)	7 (17.50%)	0.371
Epistaxis	47 (7.69%)	3 (7.50%)	1.000
Hyposmia	30 (4.91%)	0 (0.00%)	0.247
Septal perforation	23 (3.76%)	1 (2.50%)	1.000
DVT	4 (0.65%)	0 (0.00%)	1.000
Vascular injury	1 (0.16%)	1 (2.50%)	0.119
30-day neurosurgical readmission	7 (1.15%)	2 (5.00%)	0.101
Postop radiation	27 (4.42%)	3 (7.50%)	0.421
Tumor recurrence	29 (4.75%)	4 (10.00%)	0.137
Follow up (days), median, IQR	372 [57.5–716]	365 [31–910]	0.872

Bold entries signify statistical significance, *p* < 0.05

Preop, Preoperative; BMI, Body mass index; intraop, Intraoperative; KPS, Karnofsky performance score; hx, History; VD, Visual disturbances; TV, tumor volume; EOR, extent of resection; LOS, length of stay; postop, postoperative; DI, diabetes insipidus; DVT, deep vein thrombosis

Regarding operative characteristics, there were no significant difference in intraoperative CSF leaks and use of lumbar drains between groups. Uninsured patients experienced significantly lower EOR (85.44 ± 26.03% vs. 96.46 ± 9.60%, *p* = 0.017). Following surgery, uninsured patients experienced longer LOS (5 [3–7] vs. 3 [3–4] days, *p* ≤ 0.001). While there was an increased rate of overall complications (67.50% vs. 57.93%, *p* = 0.306), postoperative CSF leak (5.00% vs. 3.27%, *p* = 0.639) and hyponatremia (17.50% vs. 11.46%, *p* = 0.371) in uninsured group, the difference was not statistically significant. There were no differences in other postoperative complications, 30-day neurosurgical readmission, or follow up between groups (Table 1). While there was a trend towards higher rate of tumor recurrences in the uninsured group (10.00% vs. 4.75%), the difference was not statistically significant (*p* = 0.137).

### Insurance status: regression analysis

**Fig. 1:**
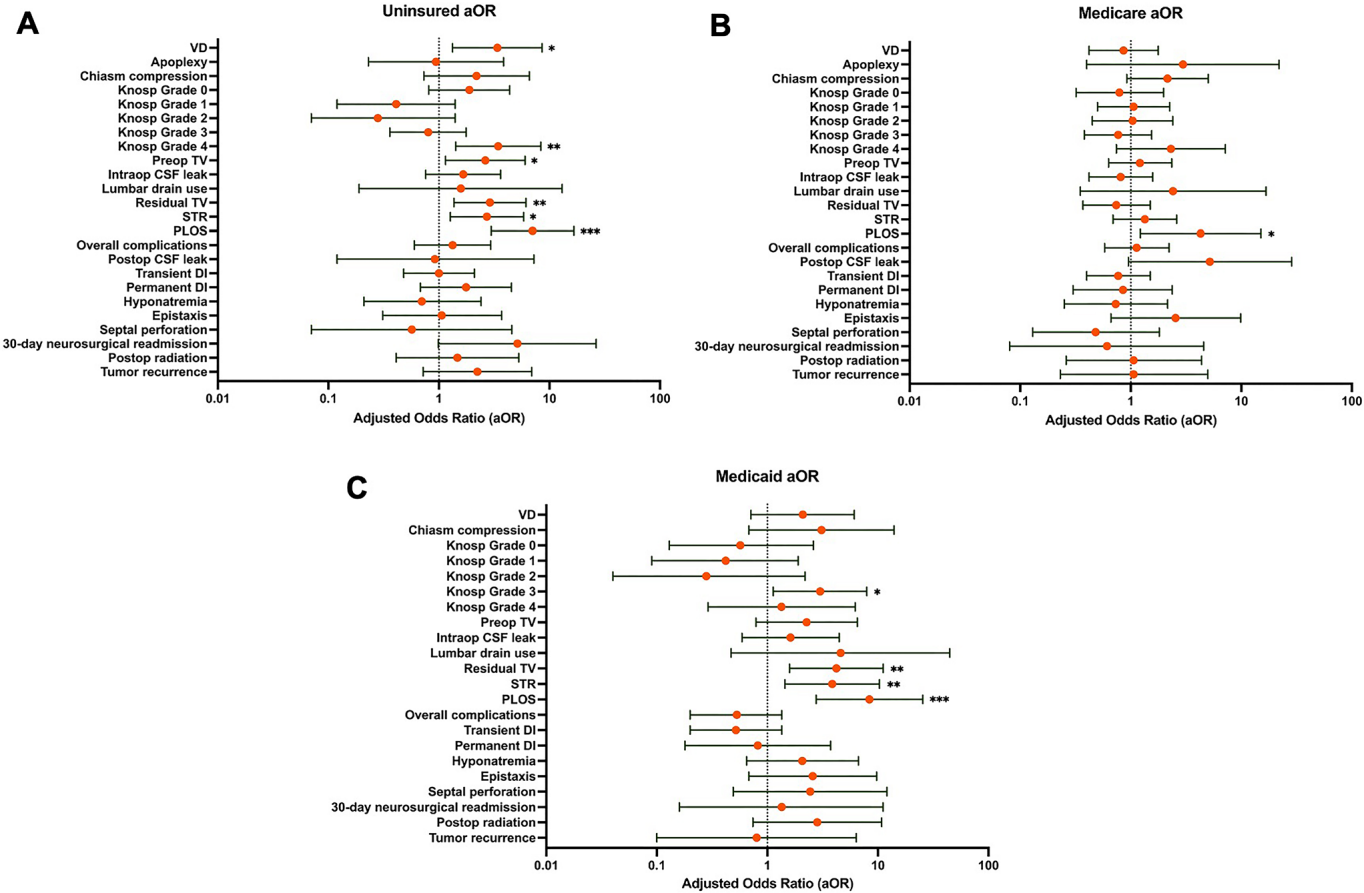
Forest plots with adjusted odds ratios of variables associated with **A**) uninsured, **B**) medicare, and **C**) medicaid group. *p < 0.05, **p < 0.01, and ***p < 0.001

Univariate logistic regression revealed that patients with greater age were less likely to be uninsured (OR 0.25 [0.10, 0.58]; *p* = 0.001). Patients with lower KPS, VD, Knosp 4 grade, higher preoperative TV, STR, and PLOS were more likely to be uninsured (*p* < 0.05). Upon multivariate regression controlling for age, gender, and preoperative KPS, VD (aOR 3.38 [1.33, 8.59]; *p* = 0.010), higher preoperative TV (aOR 2.63 [1.15, 6.01]; *p* = 0.022), Knosp 4 grade (aOR 3.44 [1.42, 8.36]; *p* = 0.006), STR (aOR 2.72 [1.27, 5.84]; *p* = 0.010), and PLOS (aOR 7.03 [2.97, 16.63]; *p* ≤ 0.001) were significantly associated with uninsured status (Table 2; Fig. 1A).

**Table 2:** Univariate and multivariate logistic regression of factors associated with lack of insurance

Variable (ref: Insured)	Uninsured			
	OR (95% CI)	*P* value	aOR (95% CI)	*P* value
**Patient demographics:**				
Age (> median)	0.25 (0.10, 0.58)	**0.001**		
Gender, male	0.86 (0.42,1.76)	0.678		
Preop KPS (< median)	2.18 (1.07,4.46)	**0.034**		
VD	3.32 (1.34,8.20)	**0.009**	3.38 (1.33, 8.59)	**0.010**
Apoplexy	1.77 (0.51, 6.18)	0.371	0.94 (0.23, 3.84)	0.925
**Adenoma characteristics**:				
Chiasm compression	2.09 (0.72, 6.07)	0.178	2.19 (0.73, 6.58)	0.163
Knosp grades:				
Grade 0	2.03 (0.91, 4.55)	0.086	1.89 (0.81, 4.37)	0.139
Grade 1	0.37 (0.11, 1.22)	0.102	0.41 (0.12, 1.40)	0.155
Grade 2	0.32 (0.07, 1.35)	0.120	0.28 (0.07, 1.22)	0.090
Grade 3	0.85 (0.39, 1.83)	0.669	0.80 (0.36, 1.76)	0.579
Grade 4	3.21 (1.37, 7.56)	**0.008**	3.44 (1.42, 8.36)	**0.006**
Preop TV (cm^3^) (> median)	2.65 (1.20, 5.85)	**0.016**	2.63 (1.15, 6.01)	**0.022**
**Operative characteristics**:				
Intraop CSF leak	1.73 (0.80, 3.72)	0.164	1.66 (0.76, 3.62)	0.205
Lumbar drain	1.28 (0.16, 10.18)	0.814	1.58 (0.19, 13.04)	0.671
Postop TV (cm^3^) (> median)	2.65 (1.29, 5.45)	**0.008**	2.89 (1.37, 6.14)	**0.006**
STR	2.59 (1.26, 5.32)	**0.010**	2.72 (1.27, 5.84)	**0.010**
**Treatment outcomes**:				
PLOS	8.31 (3.66, 18.87)	**< 0.001**	7.03 (2.97, 16.63)	**< 0.001**
Overall complications	1.47 (0.68, 3.18)	0.325	1.33 (0.60, 2.94)	0.480
Postop CSF leak	0.90 (0.12, 6.95)	0.916	0.92 (0.12, 7.23)	0.934
Transient DI	1.16 (0.60, 2.38)	0.678	1.00 (0.48, 2.09)	0.996
Permanent DI	1.62 (0.64, 4.09)	0.308	1.76 (0.68, 4.54)	0.242
Hyponatremia	0.73 (0.22, 2.46)	0.606	0.70 (0.21, 2.41)	0.576
Epistaxis	1.05 (0.31, 3.57)	0.942	1.06 (0.31, 3.69)	0.924
Hyposmia	NA	NA	NA	NA
Septal perforation	0.76 (0.10, 5.82)	0.789	0.57 (0.07, 4.57)	0.596
DVT	NA	NA	NA	NA
Vascular injury	NA	NA	NA	NA
30-day neurosurgical readmission	4.59 (0.92, 23.07)	0.064	5.13 (0.99, 26.47)	0.051
Postop radiation	1.84 (0.53, 6.45)	0.339	1.47 (0.41, 5.29)	0.554
Tumor recurrence	2.54 (0.83, 7.80)	0.102	2.23 (0.72, 6.92)	0.168

Bold entries signify statistical significance, *p* < 0.05

Preop, Preoperative; KPS, Karnofsky performance score; VD, Visual disturbances; TV, Tumor volume; intraop, Intraoperative; postop, Postoperative; STR, Subtotal resection; PLOS, Prolonged length of stay; DI, Diabetes insipidus; DVT, Deep vein thrombosis

### Insurance subgroups: descriptive statistics

Insured patients were subcategorized based on their insurance providers into Private (*n* = 361, mean age 48.77 ± 12.01 years, 50.41% male), Medicare (*n* = 223, mean age 72.22 ± 7.48 years, 57.40% male), and Medicaid (*n* = 23, mean age 50.70 ± 15.52 years, 47.83% male). There was no significant difference in gender, BMI, smoking status, history of radiation, and apoplexy between groups (Table 3). Patients with private insurance had lower rates of diabetes mellitus and hypertension. While Medicaid group had lower preoperative KPS and higher rates of VD than Private and Medicare, the differences were not statistically significant. Moreover, Medicaid patients had higher preoperative TVs (*p* = 0.006), higher rates of chiasm compression (*p* = 0.007), and Knosp 3 grade (*p* = 0.014).

There were no differences in operative characteristics between insured groups (Table 3). Postoperatively, patients with Medicaid insurance experienced longer LOS compared to those with private insurance and Medicare (5 [3–6] vs. 3 [3–3] vs. 3 [3–4] days respectively, *p* ≤ 0.001). There was an increase in overall complications and transient DI in Private group and DVT in Medicare group (*p* < 0.05). However, there were no differences in other postoperative complications, 30-day neurosurgical readmissions or tumor recurrence between groups.

**Table 3:** Patient demographics, clinical, and perioperative characteristics for insured subgroup

Variable	Private = 361	Medicare = 223	Medicaid = 23	*p* value
**Patient demographics:**				
Age (years), mean, SD	48.77 ± 12.01 a	72.22 ± 7.48 a, b	50.70 ± 15.52 b	**< 0.001**
Gender, male	182 (50.41%)	128 (57.40%)	11 (47.83%)	0.230
Preop KPS, median, IQR	90 (80–90)	90 (80–90)	80 (80–90)	0.097
BMI, mean, SD	29.98 ± 6.53	28.71 ± 5.34	29.43 ± 6.36	0.043
Diabetes mellitus	87 (24.10%)	77 (34.53%)	9 (39.13%)	**0.013**
Hypertension	162 (44.87%)	150 (67.26%)	14 (60.87%)	**< 0.001**
Active smoker at time of surgery	23 (6.37%)	20 (8.97%)	3 (13.04%)	0.227
Hx of radiation	4 (1.11%)	2 (0.90%)	0 (0.00%)	1.000
VD	194 (53.74%)	129 (57.85%)	17 (73.91%)	0.132
Apoplexy	29 (8.03%)	9 (4.04%)	0 (0.00%)	0.085
**Adenoma characteristics**:				
Chiasm compression	256 (71.11%)	182 (81.61%)	20 (86.96%)	**0.007**
Macroadenoma	337 (93.61%)	213 (95.52%)	22 (95.65%)	0.658
Knosp grades:				
Grade 0	66 (18.33%)	30 (13.45%)	2 (8.70%)	0.210
Grade 1	78 (21.67%)	54 (24.22%)	3 (13.04%)	0.451
Grade 2	62 (17.22%)	46 (20.63%)	2 (8.70%)	0.323
Grade 3	129 (35.83%)	69 (30.94%)	14 (60.87%)	**0.014**
Grade 4	25 (6.93%)	24 (10.76%)	2 (8.70%)	0.238
Preop TV (cm^3^), mean, SD	6.25 ± 9.56 a	6.43 ± 7.19	14.28 ± 28.82 a	**0.006**
**Operative characteristics**:				
Intraop CSF leak	194 (53.89%)	121 (54.26%)	15 (65.22%)	0.570
Lumbar drain	5 (1.39%)	7 (3.14%)	1 (4.35%)	0.178
Residual TV (cm^3^), mean, SD	0.34 ± 1.53 a	0.35 ± 1.19 b	0.66 ± 1.32 a, b	0.559
EOR (%), mean, SD	96.81 ± 9.58 a	96.17 ± 9.55 b	93.21 ± 10.88 a, b	0.192
**Treatment outcomes**:				
LOS (days), median, IQR	3 (3–3) a, c	3 (3–4) b, c	5 (3–6) a, b	**< 0.001**
Overall complications	230 (63.91%)	111 (49.78%)	11 (47.83%)	**0.003**
Postop CSF leak	13 (3.61%)	7 (3.14%)	0 (0.00%)	0.918
Transient DI	195 (54.17%)	74 (33.18%)	9 (39.13%)	**< 0.001**
Permanent DI	39 (10.83%)	25 (11.21%)	2 (8.70%)	0.971
Hyponatremia	41 (11.31%)	23 (10.31%)	5 (21.74%)	0.259
Epistaxis	25 (6.94%)	19 (8.52%)	3 (13.04%)	0.398
Hyposmia	14 (3.89%)	14 (6.28%)	2 (8.70%)	0.215
Septal perforation	15 (4.16%)	7 (3.14%)	1 (4.35%)	0.674
DVT	0 (0.00%)	4 (1.79%)	0 (0.00%)	**0.033**
Vascular injury	0 (0.00%)	1 (0.45%)	0 (0.00%)	0.406
30-day neurosurgical readmission	2 (0.56%)	5 (2.24%)	0 (0.00%)	0.200
Postop radiation	18 (5.00%)	6 (2.69%)	3 (13.04%)	0.052
Tumor recurrence	19 (5.28%)	9 (4.04%)	1 (4.35%)	0.137
Follow up (days), median, IQR	373 [81.75–727]	366.5 [41–694]	312 [35.5-526.5]	0.171

a, b, c denote significance upon post-hoc analysis

Preop, Preoperative; BMI, Body mass index; intraop, Intraoperative; KPS, Karnofsky performance score; hx, History; VD, Visual disturbances; TV, Tumor volume; EOR, Extent of resection; LOS, Length of stay; postop, Postoperative; DI, Diabetes insipidus; DVT, Deep vein thrombosis

### Insurance subgroups: regression analysis

On multivariate analysis, patients with Knosp 3 grade (aOR 3.00 [1.13, 7.95]; *p* = 0.027), STR (aOR 3.86 [1.44, 10.34]; *p* = 0.007), and PLOS (aOR 8.38 [2.76, 25.48]; *p* < 0.001) were more likely to have Medicaid insurance. Patients with Medicare had higher odds of PLOS as well (aOR 4.28 [1.22, 15.03]; *p* = 0.023) (Table 4; Fig. 1B and C).

**Table 4:** Multivariate logistic regression of factors associated with Medicare and Medicaid insurance

Variable (ref: Private)	Medicare		Medicaid	
	aOR (95% CI)	*P* value	aOR (95% CI)	*P* value
**Patient demographics:**				
VD	0.86 (0.42, 1.77)	0.683	2.09 (0.71, 6.11)	0.180
Apoplexy	2.97 (0.40, 21.98)	0.287	NA	NA
**Adenoma characteristics**:				
Chiasm compression	2.15 (0.92, 5.02)	0.076	3.09 (0.68, 14.00)	0.144
Knosp grades:				
Grade 0	0.79 (0.32, 1.98)	0.617	0.57 (0.13, 2.61)	0.468
Grade 1	1.06 (0.50, 2.25)	0.884	0.42 (0.09, 1.90)	0.260
Grade 2	1.04 (0.45, 2.40)	0.928	0.28 (0.04, 2.19)	0.227
Grade 3	0.77 (0.38, 1.55)	0.460	3.00 (1.13, 7.95)	**0.027**
Grade 4	2.31 (0.74, 7.19)	0.150	1.34 (0.29, 6.23)	0.712
Preop TV (cm^3^) (> median)	1.21 (0.63, 2.35)	0.565	2.27 (0.79, 6.51)	0.129
**Operative characteristics**:				
Intraop CSF leak	0.81 (0.42, 1.58)	0.536	1.62 (0.59, 4.46)	0.347
Lumbar drain	2.41 (0.35, 16.73)	0.375	4.60 (0.47, 44.74)	0.189
Postop TV (cm^3^) (> median)	0.74 (0.37, 1.50)	0.406	4.21 (1.59, 11.17)	**0.004**
STR	1.34 (0.69, 2.60)	0.381	3.86 (1.44, 10.34)	**0.007**
**Treatment outcomes**:				
PLOS	4.28 (1.22, 15.03)	**0.023**	8.38 (2.76, 25.48)	**< 0.001**
Overall complications	1.13 (0.58, 2.22)	0.720	0.53 (0.20, 1.35)	0.180
Postop CSF leak	5.20 (0.95, 28.63)	0.058	NA	
Transient DI	0.77 (0.40, 1.50)	0.440	0.52 (0.20, 1.35)	0.178
Permanent DI	0.85 (0.30, 2.37)	0.754	0.82 (0.18, 3.73)	0.797
Hyponatremia	0.73 (0.25, 2.16)	0.574	2.07 (0.65, 6.66)	0.221
Epistaxis	2.54 (0.66, 9.86)	0.178	2.57 (0.68, 9.72)	0.164
Hyposmia	0.48 (0.13, 1.81)	0.281	2.44 (0.49, 12.07)	0.276
Septal perforation	0.61 (0.08, 4.56)	0.627	1.35 (0.16, 11.12)	0.780
DVT	NA	NA	NA	NA
Vascular injury	NA	NA	NA	NA
30-day neurosurgical readmission	NA	NA	NA	NA
Postop radiation	1.06 (0.26, 4.39)	0.937	2.83 (0.74, 10.83)	0.128
Tumor recurrence	1.06 (0.23, 4.97)	0.943	0.80 (0.10, 6.39)	0.836

Bold entries signify statistical significance, *p* < 0.05

Preop, Preoperative; KPS, Karnofsky performance score; VD, Visual disturbances; TV, Tumor volume; intraop, Intraoperative; postop, Postoperative; STR, Subtotal resection; PLOS, Prolonged length of stay; DI, Diabetes insipidus; DVT, Deep vein thrombosis

## Discussion

Traditionally, employer-sponsored insurance has been the backbone of the US health coverage system, restricting healthcare access to those with the ability to work full-time [19]. As a result, older Americans and those with low incomes often did not have health insurance. In 1965, the Medicare and Medicaid programs were established in the US, extending health coverage to elderly, low-income, and disabled Americans [20]. Despite these legislative efforts, 7.9% of the US population remains uninsured [21]. These patients often have limited access to healthcare outside of emergency services [22], leading to more severe disease at the time of presentation as noted across both neurosurgical and non-neurosurgical fields [14, 23–25]. Moreover, individuals with Medicaid often experience issues with the consistency and adequacy of care due to variations in state-level Medicaid programs [26].

### Study overview

In this study, we found that uninsured patients presented with more advanced disease, had higher preoperative TV leading to reduced EOR, and increased LOS compared to insured counterparts. Patients who experienced lower preoperative KPS, increased VD, preoperative TV, Knosp 4 grade, STR, and PLOS were more likely to be uninsured.

Upon subgroup analysis, Medicaid patients had increased preoperative TV, presented in advanced stage with associated comorbidities, and experienced longer LOS compared to non-Medicaid patients. Those who experienced Knosp 3 grade, STR, and PLOS were more likely to have Medicaid insurance compared to those with private insurance.

### Patient presentation

Uninsured patients had a significantly lower preoperative KPS (85 [80–90] vs. 90 [80–90]; *p* = 0004). While Medicaid patients had lower preoperative KPS compared to Medicare and Private (80 [80–90] vs. 90 [80–90] vs. 90 [80–90]), this difference was not statistically significant. To our knowledge, there is no other literature analyzing differences in preoperative KPS by insurance status or type.

VD is one of the most prominent presentations of NFPA, with a recent meta-analysis on 35 case series reporting the incidence ranging from 28 to 100% [27]. In our study, uninsured patients were more likely to present with VD (82.50% vs. 55.81%; *p* = 0.002). On subgroup analysis of insured patients, Medicaid patients were more likely to present with VD than Private and Medicare patients (73.91% vs. 53.74% vs. 57.85%, respectively), although the difference did not reach statistical significance. This may be due to limited access to early care, leading to delayed presentation with more advanced symptoms. Similar to our findings, Osorio et al. noted a higher prevalence of VD among Medicaid patients compared to Private and Medicare (62.5% vs. 46.7% vs. 44.2%, respectively). However, they also failed to achieve statistical significance [10]. Contrary to Osorio et al. and our findings, Younus et al., including both NFPA and FPA in their cohorts, found no differences in rates of VD among Medicaid and non-Medicaid patients (44% vs. 43%, respectively) [13].

We also noted an increasing trend of apoplexy in uninsured patients (10.00% vs. 6.20%; *p* = 0.317). Jahangiri et al. previously reported that lack of insurance was significantly associated with pituitary apoplexy [28]. In their study, 11.85% of patients were uninsured as compared to 6.13% in our study. We believe that since our study had a smaller proportion of uninsured patients, the difference in rates of apoplexy did not reach statistical significance. Moreover, upon subgroup analysis, our results were similar to Younus et al. who did not show a statistically significant difference in rates of apoplexy among Medicaid and non-Medicaid groups [13]. To our knowledge, there are no other studies on rates of apoplexy within patients with different types of insurance providers.

### Adenoma characteristics

Our study used the ellipsoid formula to calculate tumor volume based on the maximum dimensions in axial, coronal, and sagittal planes. Although we recognize that volume segmentation software could yield more precise measurements, we lacked segmentation-based volume data in our dataset. However, as a natural sequela to delayed presentation, we noted that uninsured patients were more likely to have larger TV(13.68 ± 16.89 vs. 6.62 ± 10.27 cm³, *p* = 0.005) with significantly higher rates of chiasm compression and Knosp 4 grade. Similarly, on subgroup analysis, Medicaid patients had larger TV compared to Private and Medicare groups (14.28 ± 28.82 vs. 6.25 ± 9.56 vs. 6.43 ± 7.19 cm³, respectively), higher rates of chiasm compression, and Knosp 3 grade. There is a lack of literature regarding tumor characteristics among insured and uninsured patients. However, literature combining both FPAs and NFPAs noted that Medicaid patients had larger mean tumor diameters (26.1 mm vs. 23.1 mm) [13]. Regarding the severity of cavernous sinus invasion, in studying patients with NFPA, Osorio et al. noted higher rates of cavernous sinus invasion in Medicaid patients compared to Private and Medicare patients (62.5% vs. 33.7% vs. 43.0%, respectively; *p* = 0.009) [10]. Younus et al., with both FPAs and NFPAs in their study, failed to show a significant difference in cavernous sinus invasion between Medicaid and non-Medicaid groups [13]. This may be since 27% of patients in their study had FPAs which typically present as microadenomas. Keeping in mind a residual tumor doubling time of 3.4 years [29], it could be extrapolated that patients lacking insurance and Medicaid roughly present with a 3-4-year delay compared to their insured counterparts.

### EOR

The lower EOR in uninsured patients (85.44 ± 26.03% vs. 96.46 ± 9.60%) is likely related to greater Knosp 4 grade in those patients. This aligns with previous studies associating higher grades of cavernous sinus extension with lower EOR [30, 31]. Upon subgroup analysis, our results correlate with other studies that do not find a significant difference between EOR among patients with different insurance providers [10, 13].

### LOS

Our study uniquely demonstrates that uninsured patients have a median LOS that is 2 days longer compared to insured patients (*p* < 0.001). Similarly, the median was 2 days longer in Medicaid patients compared to Medicare and Private patients (*p* < 0.001). A previous study comparing NFPA noted that private insurance patients were discharged 1 day earlier than those with Medicare and Medicaid, although this did not reach statistical significance. With both FPAs and NFPAs in their cohort, Younus et al. noted that Medicaid patients had significantly longer LOS (9.4 vs. 3.55 days) compared to non-Medicaid patients [13]. Moreover, privately insured patients experienced shorter LOS [32]. The rate of overall complications and hyponatremia was higher in uninsured patients (67.50% vs. 57.93% and 17.50% vs. 11.46%, respectively), and this may be a factor responsible for PLOS. Among insured patients, the longer LOS in Medicaid patients could be attributable to a higher incidence of hyponatremia compared to Private and Medicare patients (21.74% vs. 11.31% vs. 10.31%). Similarly, a higher prevalence of diabetes mellitus in Medicaid patients could be a contributing factor as well [33].

### Postoperative complications

While the rates of overall complications, postoperative CSF leak, transient DI and hyponatremia were higher in uninsured group, the differences were not statistically significant. Notably, despite being significantly younger than the insured group, the uninsured group showed a trend toward higher complication rates. Further analysis using propensity score matching in future studies may help uncover statistically significant differences in complication rates related to insurance status. Subgroup analysis revealed an increased rate of overall complications and transient DI in Private group. However, when transient DI was excluded from the overall complication rate, the difference between groups was not significant anymore. Moreover, there were no differences in other specific complications between patients with different insurance providers which aligns with a previous study by Osorio et al. [10]. Younus et al., with FPAs and NFPAs into their cohort, found a statistically significant increase in postoperative complications in Medicaid group compared to non-Medicaid (14% vs. 7%; *p* < 0.05) [13]. Performing propensity score matching to further evaluate the impact of insurance on complication rate may lead to emergence of statistically significance. On the other hand, high volume facilities and surgeons with high surgical caseload have been shown to have better short-term outcomes after ETPS [34, 35], potentially equalizing our complication rates across insurance groups despite advanced disease in uninsured and Medicaid patients.

### Multivariate analysis

Lack of insurance was associated with reduced preoperative KPS, higher VD, preoperative TV, Knosp 4 grade, STR, and PLOS. Similarly, Medicaid was associated with Knosp 3 grade, STR, and PLOS (*p* < 0.05). These results are consistent with other studies in neurosurgical literature showing uninsured patients and those with Medicaid to have more severe disease upon presentation and worse postoperative outcomes [6, 12, 28, 36]. This delay in presentation may be due to reduced access to primary care physician or specialty consultation with lack of insurance and underinsurance [36, 37].

### Limitations and strengths

Our study is inherently limited by its retrospective nature. To overcome these limitations, we included only patients with complete records available. Additionally, it is a single-institution study, and our results may be influenced by the social and geopolitical setting of our institution. Therefore, larger multi-institutional studies and creation of national pituitary adenoma dataset with granular data is warranted to further investigate our findings. In our study, the probability of cavernous sinus invasion was determined using the Knosp-Steiner classification, which does not distinguish between grade 3 A and grade 3B. Future studies may consider the modified Knosp classification to enable further subgroup analysis based on these distinctions. Another limitation of our study is the absence of data on postoperative hormonal status and long-term hormone replacement needs. Future studies could help clarify the impact of insurance status on postoperative pituitary function and endocrine recovery.

Despite these limitations, our study provides valuable insights into the relationship between insurance and healthcare outcomes in patient undergoing ETPS for NFPA. Unlike existing generic database studies, we were able to collect detailed information on preoperative characteristics, tumor features, and postoperative outcomes, which are not always available in broader datasets. This granularity allows us to highlight significant healthcare disparities driven by insurance status, revealing how these factors contribute to delayed presentation, advanced disease, and prolonged length of stay.

## Conclusion

Our findings reveal substantial disparities, with uninsured and Medicaid patients presenting with more advanced disease, higher rates of STR, and experiencing PLOS compared to their insured counterparts. These disparities highlight the critical role that socioeconomic factors play in healthcare access and quality, reaffirming the need for targeted interventions to address these inequities.

## Data Availability

No datasets were generated or analysed during the current study.

## References

[1] Glauser G, Detchou DK, Dimentberg R, Ramayya AG, Malhotra NR (2021) Social Determinants of Health and Neurosurgical outcomes: current state and future directions. Neurosurg Apr 15(5):E383–e390. 10.1093/neuros/nyab030 10.1093/neuros/nyab030 33677591

[2] Hoh BL, Rabinov JD, Pryor JC, Carter BS, Barker FG 2 (2003) In-hospital morbidity and mortality after endovascular treatment of unruptured intracranial aneurysms in the United States, 1996–2000: effect of hospital and physician volume. AJNR Am J Neuroradiol 24(7):1409–1420 PMC7973658 12917139

[3] Hackett AM, Adereti CO, Walker AP et al (2024) Racial and socioeconomic status among a Patient Population presenting with Aneurysmal Subarachnoid Hemorrhage versus Unruptured Intracranial Aneurysm: a single-center study. Brain Sci Apr 18(4). 10.3390/brainsci14040394 10.3390/brainsci14040394 PMC11047834 38672043

[4] Gautam D, Findlay MC, Karsy M (2024) Socioeconomic and racial disparities affect Access to high-volume centers during Meningioma Treatment. World Neurosurg Jul 187:e289–e301. 10.1016/j.wneu.2024.04.076 10.1016/j.wneu.2024.04.076 38642832

[5] Snyder RA, Chang GJ (2020) Insurance Status as a Surrogate for Social Determinants of Health in Cancer Clinical Trials. JAMA Netw Open 3(4):e203890. 10.1001/jamanetworkopen.2020.3890 10.1001/jamanetworkopen.2020.3890 32352526

[6] Curry WT Jr., Carter BS, Barker FG 2 (2010) Racial, ethnic, and socioeconomic disparities in patient outcomes after craniotomy for tumor in adult patients in the United States, 1988–2004. Neurosurg Mar 66(3):427–437 discussion 437-8. 10.1227/01.Neu.0000365265.10141.8e 10.1227/01.NEU.0000365265.10141.8E 20124933

[7] El-Sayed AM, Ziewacz JE, Davis MC et al (2011) Insurance status and inequalities in outcomes after neurosurgery. World Neurosurg Nov 76(5):459–466. 10.1016/j.wneu.2011.03.051 10.1016/j.wneu.2011.03.051 22152576

[8] Daly AF, Beckers A (2020) The epidemiology of Pituitary Adenomas. Endocrinol Metab Clin North Am Sep 49(3):347–355. 10.1016/j.ecl.2020.04.002 10.1016/j.ecl.2020.04.002 32741475

[9] Olsson DS, Svensson M, Labori F, De Geer A, Johannsson G (2023) Healthcare cost and survival in patients with non-functioning pituitary adenoma. Eur J Endocrinol Jun 7(6):477–484. 10.1093/ejendo/lvad057 10.1093/ejendo/lvad057 37232269

[10] Osorio RC, Pereira MP, Joshi RS et al (2022) Socioeconomic predictors of case presentations and outcomes in 225 nonfunctional pituitary adenoma resections. J Neurosurg May 1(5):1325–1336. 10.3171/2021.4.Jns21907 10.3171/2021.4.JNS21907 34598141

[11] Goljo E, Parasher AK, Iloreta AM, Shrivastava R, Govindaraj S (2016) Racial, ethnic, and socioeconomic disparities in pituitary surgery outcomes. Laryngoscope Apr 126(4):808–814. 10.1002/lary.25771 10.1002/lary.25771 26845457

[12] McKee S, Yang A, Kidwai S, Govindaraj S, Shrivastava R, Iloreta A (2018) The socioeconomic determinants for transsphenoidal pituitary surgery: a review of New York State from 1995 to 2015. Int Forum Allergy Rhinol Oct 8(10):1145–1156. 10.1002/alr.22148 10.1002/alr.22148 30007017

[13] Younus I, Gerges M, Schwartz TH, Ramakrishna R (2020) Impact of Medicaid insurance on outcomes following endoscopic transsphenoidal pituitary surgery. J Neurosurg Mar 20(3):801–806. 10.3171/2020.1.Jns192707 10.3171/2020.1.JNS192707 32197242

[14] Jahangiri A, Lamborn KR, Blevins L, Kunwar S, Aghi MK (2012) Factors associated with delay to pituitary adenoma diagnosis in patients with visual loss. J Neurosurg Feb 116(2):283–289. 10.3171/2011.6.Jns101663 10.3171/2011.6.JNS101663 21740118

[15] Gaffney A, McCormick D (2017) The affordable Care Act: implications for health-care equity. Lancet Apr 8(10077):1442–1452. 10.1016/s0140-6736(17)30786-9 10.1016/S0140-6736(17)30786-9 28402826

[16] Chen SH, Sprau A, Chieng L et al (2019) Transsphenoidal Approach for Pituitary adenomas in Elderly patients. World Neurosurg Jan 121:e670–e674. 10.1016/j.wneu.2018.09.187 10.1016/j.wneu.2018.09.187 30292662

[17] Ghiam MK, Ali IA, Dable CL et al (2022) Multidisciplinary postoperative care pathway to reduce readmissions following endoscopic transsphenoidal pituitary surgery: improving Quality of Patient Care. J Neurol Surg B Skull Base Dec 83(6):626–634. 10.1055/a-1920-0758 10.1055/a-1920-0758 PMC9653288 36393882

[18] Vimawala S, Chitguppi C, Reilly E et al (2020) Predicting prolonged length of stay after endoscopic transsphenoidal surgery for pituitary adenoma. Int Forum Allergy Rhinol Jun 10(6):785–790. 10.1002/alr.22540 10.1002/alr.22540 32362064

[19] Corlette S, Monahan CH (2022) U.S. Health Insurance Coverage and Financing. N Engl J Med Dec 22(25):2297–2300. 10.1056/NEJMp2206049 10.1056/NEJMp2206049 36547724

[20] Berkowitz E (2008) Medicare and Medicaid: the past as prologue. Health Care Financ Rev Spring 29(3):81–93 PMC4195036 18567245

[21] Health Insurance Coverage in the United States (2022) (U.S. Government Publishing Office) (2023)

[22] Pitts SR, Carrier ER, Rich EC, Kellermann AL (Sep 2010) Where Americans get acute care: increasingly, it’s not at their doctor’s office. Health Aff (Millwood) 29(9):1620–1629. 10.1377/hlthaff.2009.1026 10.1377/hlthaff.2009.1026 20820017

[23] He G, Li C, Wang S, Wang H, Ding J (2022) Association of insurance status with chronic kidney disease stage at diagnosis in children. Pediatr Nephrol Nov 37(11):2705–2714. 10.1007/s00467-022-05493-6 10.1007/s00467-022-05493-6 35224660

[24] Markt SC, Lago-Hernandez CA, Miller RE et al (2016) Insurance status and disparities in disease presentation, treatment, and outcomes for men with germ cell tumors. Cancer Oct 15(20):3127–3135. 10.1002/cncr.30159 10.1002/cncr.30159 PMC5048492 27500561

[25] Farley TA, Flannery JT (1989) Late-stage diagnosis of breast cancer in women of lower socioeconomic status: public health implications. Am J Public Health Nov 79(11):1508–1512. 10.2105/ajph.79.11.1508 10.2105/ajph.79.11.1508 PMC1349803 2817162

[26] Ramírez de Arellano A (2007) A ranking of State Medicaid Programs. Public Citizen Health Research Group. https://www.citizen.org/article/unsettling-scores/

[27] Muskens IS, Zamanipoor Najafabadi AH, Briceno V et al (2017) Visual outcomes after endoscopic endonasal pituitary adenoma resection: a systematic review and meta-analysis. Pituit Oct 20(5):539–552. 10.1007/s11102-017-0815-9 10.1007/s11102-017-0815-9 PMC5606952 28643208

[28] Jahangiri A, Clark AJ, Han SJ, Kunwar S, Blevins LS Jr., Aghi MK (2013) Socioeconomic factors associated with pituitary apoplexy. J Neurosurg Dec 119(6):1432–1436. 10.3171/2013.6.Jns122323 10.3171/2013.6.JNS122323 23889139

[29] Chen Y, Wang CD, Su ZP et al (2012) Natural history of postoperative nonfunctioning pituitary adenomas: a systematic review and meta-analysis. Neuroendocrinology 96(4):333–342. 10.1159/000339823 22687984 10.1159/000339823

[30] Serra C, Staartjes VE, Maldaner N et al (2018) Predicting extent of resection in transsphenoidal surgery for pituitary adenoma. Acta Neurochir (Wien) Nov 160(11):2255–2262. 10.1007/s00701-018-3690-x 10.1007/s00701-018-3690-x 30267209

[31] Singla R, Sharma R, Suri A (2023) Role of cavernous sinus extension and MRI T2 hypointensity in the extent of resection following trans-sphenoidal surgery for Giant Pituitary Adenomas. Neurol India 71(5):907–915. 10.4103/0028-3886.388120 10.4103/0028-3886.388120 37929426

[32] Hamill CS, Villwock JA, Sykes KJ, Chamoun RB, Beahm DD (2018) Socioeconomic factors affecting discharge status of patients with uncomplicated Transsphenoidal Adenohypophysectomy. J Neurol Surg B Skull Base Oct 79(5):501–507. 10.1055/s-0038-1635095 10.1055/s-0038-1635095 PMC6133663 30210979

[33] Sharma A, Muir R, Johnston R, Carter E, Bowden G, Wilson-MacDonald J (2013) Diabetes is predictive of longer hospital stay and increased rate of complications in spinal surgery in the UK. Ann R Coll Surg Engl May 95(4):275–279. 10.1308/003588413x13511609958299 10.1308/003588413X13511609958299 PMC4132503 23676813

[34] Chalif EJ, Couldwell WT, Aghi MK (2022) Effect of facility volume on giant pituitary adenoma neurosurgical outcomes. J Neurosurg Sep 1(3):658–667. 10.3171/2021.11.Jns211936 10.3171/2021.11.JNS211936 35171824

[35] Barker FG 2nd, Klibanski A, Swearingen B (2003) Transsphenoidal surgery for pituitary tumors in the United States, 1996–2000: mortality, morbidity, and the effects of hospital and surgeon volume. J Clin Endocrinol Metab Oct 88(10):4709–4719. 10.1210/jc.2003-030461 10.1210/jc.2003-030461 14557445

[36] Dasenbrock HH, Wolinsky JP, Sciubba DM, Witham TF, Gokaslan ZL, Bydon A (2012) The impact of insurance status on outcomes after surgery for spinal metastases. Cancer 118(19):4833-41. 10.1002/cncr.27388 10.1002/cncr.27388 22294322

[37] DeSantis C, Jemal A, Ward E (2010) Disparities in breast cancer prognostic factors by race, insurance status, and education. Cancer Causes Control Sep 21(9):1445–1450. 10.1007/s10552-010-9572-z 10.1007/s10552-010-9572-z 20506039

